# Is Chlorhexidine in Dentistry an Ally or a Foe? A Narrative Review

**DOI:** 10.3390/healthcare10050764

**Published:** 2022-04-20

**Authors:** Łukasz Pałka, Agnieszka Nowakowska-Toporowska, Bartosz Dalewski

**Affiliations:** 1 Private Dental Practice, 68-200 Zary, Poland; 2 Department of Dental Prosthetics, Wroclaw Medical University, 50-367 Wroclaw, Poland; agano125@gmail.com; 3 Department of Dental Prosthetics, Pomeranian Medical University, 70-204 Szczecin, Poland; bartosz.dalewski@pum.edu.pl

**Keywords:** chlorhexidine, adverse effects, oral microbiome, disinfection, oral hygiene

## Abstract

Chlorhexidine has been one of the most effective and popular antiseptic substances used in medicine for decades. In dentistry, it has been used in endodontics, periodontology, surgery, and general dentistry. It is also widely used daily by patients in mouth rinses, gels, or toothpastes. Because of its multiple uses, we should follow all types of research reporting its potential adverse effects. This article aims to review the most up-to-date studies regarding chlorhexidine and its possible side effects, in the period of the SARS-CoV-2 pandemic, as the use of different antiseptic substances has rapidly increased.

## 1. Introduction

Chlorhexidine (CHX) is one of the most effective, and thus most popular antiseptic substances used in medicine. Its broad spectrum of application covers such fields as endodontics, periodontology, surgery, and general dentistry. It is one of the compounds of toothpastes, mouth rinses, post-operative gels, and medicinal products widely available at every chemist. Due to this, its usage is beyond any control and frequently abused.

Since the beginning, CHX gained a lot of attention from scientists. Lewis et al. suggested bathing the patient in it to prevent hospital-acquired infections [1]. Moreover, it is mentioned in the Model List of Essential Medicines (2019)—a type of recommendation list—as one of the safest and most effective medicines needed in a health system, according to the WHO guidelines. In the 1970s, thanks to its ability to inhibit the formation and development of plaque, CHX also became acknowledged in dentistry, becoming one of the most commonly used chemical agents [2]. However, one of the imperfections of human nature is a tendency to exaggerate in search of a golden mean.

Up to 2019, the number of in vitro and in vivo studies on adaptation and cross-adaptation levels for oral pathogens exposed to antiseptics, such as CHX, was surprisingly small [3]. In contrast, there were studies and reports regarding bacteria resistance and cross-resistance for commonly used antiseptics, including CHX [4]. The reason for the recent increase in papers regarding this topic has been connected with the reported emergence of Gram-negative bacteria resistant to colistin, which is a last resort antibiotic after exposure to CHX [5,6].

During the COVID-19 pandemic, as a result of a lack of effective treatment methods and problems with access to personal protective equipment, including masks or protective helmets, in order to restrict virus spreading and transmission, attention has been directed to hand disinfectants and mouth rinses [7]. The strategy with mouth rinses was to reduce the salivary load of the SARS-CoV-2 virus; thus, many substances such as chlorhexidine, povidone-iodine, essential oils, hydrogen peroxide, and cetylpyridinium chloride were analyzed [8,9,10]. Although the results were inconclusive, with very limited clinical evidence for the effectiveness of mouth rinses against SARS-CoV-2 [11], it did not discourage average citizens from supplying their homes with an antivirus arsenal of mouth rinses, especially as they were commonly available. One of the potential risks of such a situation is an uncontrolled abuse of such substances, which may have potentially harmful effects on our health if overdosed. One such potentially dangerous substance, which has been at the gunpoint of researchers over the last few years, is chlorhexidine gluconate and the COVID-19 pandemic increased its sales dramatically [12].

### Historical Perspective

Chlorhexidine was first mentioned in 1950, when a group of researchers from Imperial Chemical Industries Limited, Biological and Research Laboratories, were investigating the biological properties of polydiguanides and one compound, No. 10,040, had drawn their attention because of its outstanding antibacterial activity. After further experiments, they came to the conclusions that its antibacterial action is exerted against a wide range of Gram-positive, Gram-negative, and vegetative bacteria but reduced against bacterial spores. At the time, this substance was called “Hibitane,” but then was renamed as “Chlorhexidine” (CHX) [13]. In 1976, Emilson and Fornell reported that long-term use of CHX could lead to the development of adaptation in some bacteria species through increased minimum inhibitory concentrations (MIC) [14]. In addition, Rushton mentioned occasional oral intolerance of mouthwash containing CHX and occasional parotid gland swelling [15]. It seems that the positive effects of inhibiting plaque formation and accumulation were more important and clinically significant than its adverse effects [16]. For this reason, it is of the utmost importance for our patients and our own sakes to confront every piece of research or experiment reporting that there may be some adverse effects of using such a popular antiseptic agent. The aim of this paper is to present up-to-date research results indicating the potentially dangerous effects of misusing chlorhexidine in dentistry and prophylaxis.

## 2. Material and Methods

The search strategy was carried out by two authors independently (Ł.P. and A.N.-T.). There were no time restrictions, and it was updated to April 2022. The PubMed database was analyzed with the keywords: (chlorhexidine) AND ((adverse effects) OR (cross-resistance) OR (overdose) OR (SARS-CoV-2)). After screening of the results (2035 articles), 31 articles have been included in this narrative review. Inclusion criteria used for this narrative review were as follows: randomized controlled trials, randomized clinical trials, case-control studies, case reports, animal studies, and papers written in English. The exclusion criteria were as follows: comments, narrative reviews, and papers written in languages other than English.

## 3. Clinical Implications

The oral microbiome is one of the most sophisticated environments containing bacteria, fungi, viruses, and protozoa, organized in complex multi-structural biofilms, which are in constant interaction with each other, the host immunological system, and external factors. Maintaining balanced conditions is crucial for good health. In other words, any perturbations and distortions to this equilibrium may lead to oral and systematic diseases. Bescos et al. reported that CHX could shift the oral microbiome resulting in decreased saliva nitrate concentration, which may have a severe adverse impact on patients with high blood pressure [17]. They also demonstrated that this CHX-related microbiome shift can change the level of saliva lactate and glucose, increasing their concentration. The latter is especially important in the lately developed proteome salivary tests, in which the long-term use of CHX can theoretically give false positive results [18]. In addition, Tribble et al. reported that washing the mouth twice a day with a 0.12% CHX solution for a week significantly increased blood pressure in healthy subjects [19]. Moreover, Joshipura et al. found that those who used mouthwash twice daily had a significantly elevated risk of pre-diabetes/diabetes compared to less frequent users [20].

Changes in the microbiome and their interactions with biomaterials can also negatively affect some of their properties and, subsequently, the host response [21]. For example, lactic acid secreted by some bacteria can destroy the titanium dioxide layer crucial to the anticorrosive properties of titanium fixation plates and dental implants, contributing to their loss [22]. Chatzigiannidou et al. presented that the extensive use of CHX can change the pH of saliva and shift the microbiome population to lactic acid-producing species [23]. They also observed higher production of butyrate as a consequence of *Fusobacterium* spp. dominance, after the exposition of natural biofilm to CHX [16]. Chang et al. and Liu et al. reported that butyrate is a potent destructive factor of the gingival epithelial barrier and pro-inflammatory mediators, playing an important role in the exacerbation of periodontal disease [24,25,26].

What is more, chronic gingivitis and chronic periodontitis differ regarding the oral pH; thus, CHX can be more effective in an alkaline environment of gingivitis rather than in acidic periodontitis [17]. In vitro studies also proved that CHX negatively affected fibroblasts and osteoblasts [27,28,29]. A high dosage of CHX can negatively affect the proliferation of fibroblasts, but also very low concentrations can significantly reduce both collagen and non-collagen protein production of human gingival fibroblasts in vitro [27]. That is why using CHX gel dressing for closing or healing screws, as well as CHX gingival pocket chips, should be at least reconsidered.

According to the producers of dental products containing CHX, especially mouth rinses, the recommended time of use ranges from 2 weeks to 6 months, with a 1-month interval before reuse [30]. However, what exactly are these time frames based on, and what is the impact of the prolonged use of CHX? Below et al. used 0.2% CHX rinse three times a day for 5 days and reported a saliva concentration peak on the fourth day, which remained detectable 12 h after the last use. They also detected a high concentration of p-chloroaniline, which is potentially carcinogenic [31]. Therefore, the FDA (Federal Drug Administration) recommendation is to limit the use of CHX mouthwash to a maximum of 6 months [31]. Knowing that commercially available mouthwash solutions may contain even up to 2.5 mg of p-chloroaniline-L-chlorhexidine should be taken into consideration before recommending it to our patients [32].

When comparing all this information regarding application time with the results obtained by Verspecht et al., who studied oral photogenes’ potential to develop adaptation to CHX, this issue appears to be more serious if not alarming [3]. It seems that 10 passages are enough for the bacteria exposed to CHX to develop adaptation to chlorhexidine and cross-adaptation to antibiotics (tetracycline, azithromycin) and other antiseptic agents (cetylpyridinium chloride) [3]. We need to remember that in contrast to Verspecht’s experiment, when it comes to the daily use of CHX, the MIC (minimum inhibitory concentration) does not change. This has also been confirmed by Cieplik et al., who tried to draw the attention of researchers and clinicians to the risk of resistance to CHX in oral bacteria and the potential mechanisms conferring this resistance or even cross-resistance to antibiotics [4].

Considering all these studies, it is advisable to rethink the protocol and change the application of mouth rinses, starting from a low concentration and increasing it every week. This seems clinically reasonable as no statistically significant differences in antiseptic effectiveness were found between the groups with different CHX concentrations at 4 to 6 weeks and 6 months [29]. Recent findings on CHX should also be applied in the standard oral prophylaxis such as mouth rinsing and teeth brushing. Kolahi reported that the use of toothpaste before mouthwash with CHX would reduce staining by 18%, but when the process is reversed and CHX is applied before the dentifrice, the staining will decrease by 79% [32]. The main problem is that CHX interacts with fluoride decreasing its effectiveness; thus, it is recommended to interpose a 30-min gap between the use of products containing these substances [32]. It is worth mentioning that in the past, negative interference between CHX and sodium lauryl sulfate (SLS) dentifrice was discussed, but Elkerbout et al. showed that since it was based on the studies conducted by one scientific group, there was a high risk of partisanship [33]. In regard to plaque control and improvement of the plaque index using CHX, all the above examples have been scientifically proven [34,35,36]. On the other hand, Zanatta et al. reported that in order to increase the effectiveness of CHX in anti-plaque control and at the same time decrease its side effects, such as calculus formation and teeth staining, the treated surfaces must be plaque free [37,38].

Deschepper et al. presented the results of a comprehensive cohort study in which they found that among patients receiving chlorhexidine for oral care, the mortality rate was higher, and this was regardless of the low (<300 mg) or high (>300 mg) cumulative dosage level exposure [39]. In addition, Parreco et al. reported over a 2-year long multicenter study of 186 hospitals and conducted among 64,904 patients, in which CHX oral care appeared an independent risk factor for mortality [40]. One explanation for the higher mortality rate may be Blot’s hypothesis, according to whom one of the causes is the decreased bioavailability of nitric oxide (NO) associated with antiseptic mouthwash use [41].

Additionally, in ICUs (Intensive Care Units), performing oral hygiene procedures on ventilated patients with the use of toothbrushes or mouth rinses alone is difficult and medical personnel prefer to do it using cotton swabs or gauzes [42].

Possible alternatives to chlorhexidine-based rinses include all natural polyherbal mouthwashes [43], probiotics [44], seawater-based mouthwash [45], or Beta-Cyclodextrin + Citrox [46]; however, all these solutions need more in-depth studies.

## 4. Conclusions

CHX is active against most microbes, Gram-positive and Gram-negative organisms, facultative and strict anaerobes, aerobes, yeasts, fungi, and some viruses; the only thing it cannot kill are spores [47]. To date, the main adverse effects caused by CHX mouthwash include parotid gland swelling, pigmentation of the oral soft tissues and teeth, type 1 hypersensitivity reactions, taste alteration, burning sensation, oral mucosa ulceration or erosions, a transient anesthetic sensation, and paresthesia [48,49,50]. The effectiveness and benefits of CHX application as an antiseptic agent are supported by numerous studies and indisputable. This article deliberately focuses only on the complications and potential side effects of the uncontrolled use of CHX in dentistry and everyday life, to increase awareness that it cannot be used thoughtlessly. In the oral environment, it is not only unfavorable to eradicate all microbes because of the benefits gained from their cooperation with the microbiome but it also seems impossible because of the biofilm features. We have to remember that bacteria can generate resistance against antiseptics due to exposure to sub-lethal concentrations, and they can also develop cross-adaptation to other antiseptic substances as well as to antibiotics, and finally, dead bacteria can stimulate or modify the activity of other pathogens.

As a result of the outburst of the SARS-CoV-2 pandemic, the usage of CHX increased exceptionally; thus, in the post-pandemic period, an intensity of the serious adverse effects of overuse can be expected. Unfortunately, long-term effects require time and can accumulate, obliterating the real cause of the problem. From the clinical point of view, it is advised to increase the awareness regarding CHX not only among patients, but also among medical staff—especially in ICUs—and to consider the usage of alternative rinses and substances.

## Data Availability

Not applicable.
